# The Clinical Efficacy and Safety of Stem Cell Therapy for Diabetes Mellitus: A Systematic Review and Meta-Analysis

**DOI:** 10.14336/AD.2019.0421

**Published:** 2020-02-01

**Authors:** Yazhen Zhang, Wenyi Chen, Bing Feng, Hongcui Cao

**Affiliations:** ^1^State Key Laboratory for Diagnosis and Treatment of Infectious Diseases, the First Affiliated Hospital, College of Medicine, Zhejiang University, Hangzhou, China; ^2^Collaborative Innovation Center for Diagnosis and Treatment of Infectious Diseases, Hangzhou, China

**Keywords:** diabetes mellitus, stem cells, cell therapy, meta-analysis, regenerative medicine, systematic review

## Abstract

Diabetes mellitus (DM) is a chronic metabolic disease with high morbidity and mortality. Recently, stem cell-based therapy for DM has shown considerable promise. Here, we undertook a systematic review and meta-analysis of published clinical studies to evaluate the efficacy and safety of stem cell therapy for both type 1 DM (T1DM) and type 2 DM (T2DM). The PubMed, Cochrane Central Register of Controlled Trials, EMBASE, and ClinicalTrials.gov databases were searched up to November 2018. We employed a fixed-effect model using 95% confidence intervals (CIs) when no statistically significant heterogeneity existed. Otherwise, a random-effects statistical model was used. Twenty-one studies met our inclusion criteria: ten T1DM studies including 226 patients and eleven T2DM studies including 386 patients. Stem cell therapy improved C-peptide levels (mean difference (MD), 0.41; 95% CI, 0.06 to 0.76) and glycosylated hemoglobin (HbA1c; MD, -3.46; 95% CI, -6.01 to -0.91) for T1DM patients. For T2DM patients, stem cell therapy improved C-peptide levels (MD, 0.33; 95% CI, 0.07 to 0.59), HbA1c (MD, -0.87; 95% CI, -1.37 to -0.37) and insulin requirements (MD, -35.76; 95% CI, -40.47 to -31.04). However, there was no significant change in fasting plasma glucose levels (MD, -0.52; 95% CI, -1.38 to 0.34). Subgroup analyses showed significant HbA1c and C-peptide improvements in patients with T1DM treated with bone marrow hematopoietic stem cells (BM-HSCs), while there was no significant change in the mesenchymal stem cell (MSC) group. In T2DM, HbA1c and insulin requirements decreased significantly after MSC transplantation, and insulin requirements and C-peptide levels were significantly improved after bone marrow mononuclear cell (BM-MNC) treatment. Stem cell therapy is a relatively safe and effective method for selected individuals with DM. The data showed that BM-HSCs are superior to MSCs in the treatment of T1DM. In T2DM, MSC and BM-MNC transplantation showed favorable therapeutic effects.

According to a report from the International Diabetes Federation, there were 424.9 million diabetic patients aged 20-79 years worldwide in 2017, and the number is expected to reach 628.6 million by 2045 [1]. In addition, the number of children and adolescents (0-19 years old) with type 1 diabetes has reached 1.1 million [1]. Diabetes mellitus (DM), including type 1 DM (T1DM) and type 2 DM (T2DM), is one of the most common chronic diseases worldwide, with high morbidity and mortality rates. Although T1DM and T2DM have different pathophysiological mechanisms, including immune-inflammatory destruction of β-cells in T1DM and insulin resistance with β-cell dysfunction in T2DM [2-4], they both cause hyperglycemia and chronic multisystem complications. The complications of DM include microvascular disease (i.e., retinopathy, nephropathy, and neuropathy) and macrovascular disease (i.e., cardiovascular disease, cerebrovascular accidents, and peripheral vascular disease) [5, 6]. Insulin and other external hypoglycemic agents are often used to control high blood glucose but they cannot accurately mimic the secretion of endogenous insulin and may cause reactive hypoglycemia [7, 8]. Pancreas or islet transplantation is another alternative treatment. Whole pancreas transplantation can quickly control hyperglycemia and eliminate the need for exogenous insulin supplementation. However, drawbacks include the high morbidity associated with a major surgery, limited availability of donor pancreas, and lifelong immunosuppression and its attendant risks, including infection and malignancy [9, 10]. The rate of islet transplantation is lower than that associated with whole pancreas transplantation, but graft survival is limited and outcomes are variable, with only 10% of cases showing insulin independence after 5 years [11]. Other forms of encapsulation, immunomodulation, and delivery technologies are still under development, and remain challenging to implement [12].

Stem cells show significant therapeutic potential in patients with diabetes, due to their immunomodulatory properties and ability to regenerate into insulin-producing cells (IPCs) [13-15]. Voltarelli et al. used hematopoietic stem cells (HSCs) to treat newly diagnosed T1DM patients and reported encouraging results [16]. Bhansali et al. showed that bone marrow-derived stem cells are also a safe and effective treatment for T2DM to improve β-cell function [17]. Since then, a number of clinical studies on the treatment of T1DM and T2DM have been conducted involving bone marrow hematopoietic stem cells (BM-HSCs), mesenchymal stem cells (MSCs), and bone marrow mononuclear cells (BM-MNCs). However, it is unclear which type of stem cells is most effective in treating diabetes, and one study reported that severe infectious diseases occur after stem cell treatment [18]. Two meta-analyses explored the effects of stem cells on diabetes, but their findings were inconsistent [19, 20]. Indeed, Rahim et al. showed that stem cell therapy has a negative effect on C-peptide in patients with type 2 diabetes [19], but El-Badawy et al. found that stem cell therapy can improve C-peptide levels [20]. In addition, they studied changes in glycosylated hemoglobin (HbA1c) and C-peptide but did not systematically analyze fasting plasma glucose (FPG) levels, insulin requirements, or adverse events after stem cell therapy. We therefore undertook a systematic review and meta-analysis of clinical studies to evaluate the efficacy and safety of stem cell therapy for both T1DM and T2DM. We define safety as a lack of obvious adverse events and efficacy as a significant improvement in pancreatic endocrine function after stem cell therapy, which can be indexed by improvements in laboratory parameters such as HbA1c, C-peptide, FPG, and insulin requirements.

The objective of this systematic review and meta-analysis was to establish a summary estimate of the efficacy and safety of different types of stem cells in T1DM and T2DM. We also aimed to identify the most effective and safe cell types for T1DM and T2DM, to help optimize future stem cell treatment options for diabetes.

## MATERIALS AND METHODS

This review was performed according to the recommendations of the Preferred Reporting Items for Systematic Reviews and Meta-Analysis (PRISMA) guideline [21].

### Search strategy and selection criteria

A comprehensive search of the PubMed, Cochrane Central Register of Controlled Trials, EMBASE, and ClinicalTrials.gov databases was conducted, with no language or time restrictions until November 2018. The search strategy (see Supplementary Table 1) included the following medical keywords: “diabetes mellitus”, “hyperglycemia”, “stem cells”, “progenitor cells”, “hematopoietic stem cells”, “mesenchymal stem cells”, “bone marrow mononuclear cells”, and “cell therapy”. Filters were set for human studies and clinical trials, and the search field was set to the title or abstract. The inclusion criteria were studies with the following elements: (1) human subjects; (2) patients diagnosed with T1DM or T2DM; (3) stem cell therapy; and (4) availability of laboratory parameters for diabetes, for example, HbA1c, C-peptide, FPG, and insulin requirements. Exclusion criteria were studies on animals, reviews, conference proceedings, or studies for which the full text was unavailable. To ensure the quality of the included studies, we did not include gray literature.

### Data extraction and quality assessment

Data extraction was independently conducted by two investigators using a standardized approach to ensure accuracy of the data. Disagreement was resolved through discussion. The data collected included study information (author, year of publication, country, and follow-up period), patient demographics (number, median or mean age of patients, sex, and history of disease), cell information (regimen, number, and delivery route), and laboratory parameters for diabetes (HbA1c, C-peptide, FPG, and insulin requirements) during the follow-up period. A subgroup analysis was performed based on different stem cell types to determine the effect of cell source on the efficacy and safety of diabetes treatment.

The meta-analysis evaluated the efficacy of stem cell therapy according to the changes in laboratory parameters for diabetes after treatment. The primary study outcomes were changes in C-peptide and HbA1c levels after stem cell therapy and whether there was an adverse reaction after treatment. Changes in FPG levels and insulin requirements after stem cell therapy were considered as secondary outcomes.

Two authors independently assessed the quality of the included studies using the Downs and Black quality assessment method [22], which is appropriate for assessing both randomized and non-randomized studies. The quality of evidence was evaluated based on criteria for reporting, external validity, internal validity (bias and confounding), and power using the Downs and Black quality assessment tool. The maximum total score on the Downs and Black quality assessment tool is 32, with higher scores reflecting higher-quality research. If there was disagreement between the two authors, a consensus was reached through discussion or with assistance from a third author.

### Data analysis and statistical methods

The mean difference (MD) and related 95% confidence intervals (CIs) were calculated to evaluate the efficacy of stem cell-based therapy. All statistical analyses were conducted using the Review Manager Version 5.3 database. MD data before and after treatment were calculated using forest plots with meta-analysis in one arm. The amount of heterogeneity between studies was evaluated using the Cochrane *Q*-test (*P < 0.1* was considered to indicate significance) and the I² statistic (I² > 50% was considered to indicate high heterogeneity). We used a fixed-effect model with 95% CIs when no statistically significant heterogeneity existed. A random-effects statistical model was used when data showed significant heterogeneity. To identify heterogeneity, a sensitivity analysis with omission of one study at a time was performed. We were unable to evaluate publication bias because the sample size in each subgroup of studies was relatively small.

### Role of funding source

The funder had no role in the study design, data collection, management, analysis, or interpretation, or in the preparation or decision to publish the manuscript. All authors had full access to all research data and the corresponding author was ultimately responsible for the decision to submit the paper.

**Figure 1.**
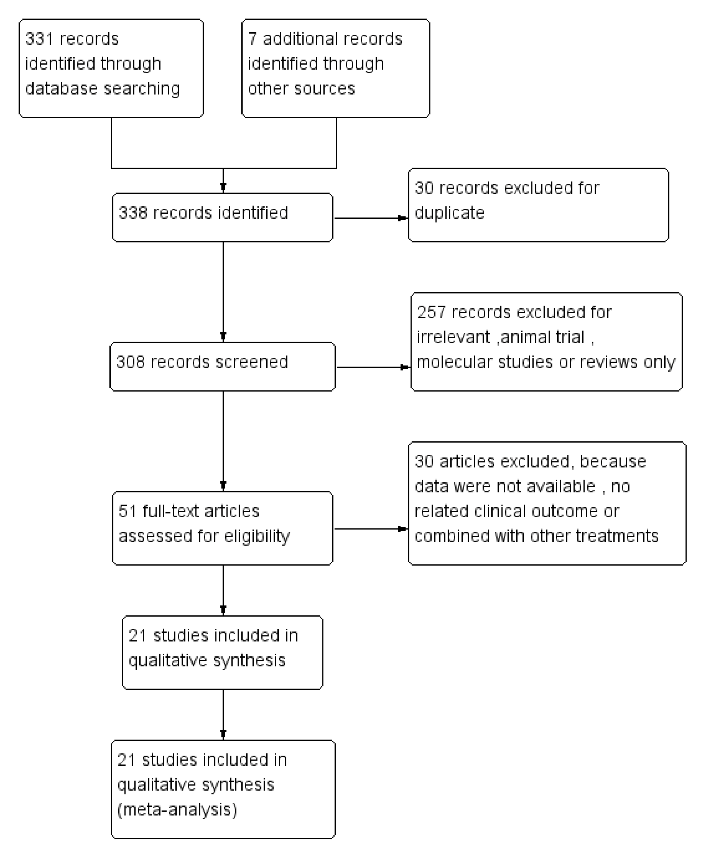
Flow chart of the selection process for this meta-analysis.

## RESULTS

### Trial selection

As shown in Figure 1, the electronic search yielded a total of 338 citations. Of these, 30 duplicated articles were excluded. After the titles and abstracts had been examined, 257 articles were excluded due to being irrelevant, or being in the form of animal trials, molecular studies, or reviews. After the full texts had been assessed, 30 articles were excluded because the data were not available, no related clinical outcomes were reported, or the outcomes were combined with other treatments. As a result, 21 eligible clinical trials reporting stem cell-based therapy for DM were included in the meta-analysis.

### Characteristics of the included studies

The characteristics of the 21 included studies are presented in Table 1. The studies were published from 2007 to 2017 and were conducted in China, Poland, Brazil, Sweden, India, and the United States. The studies included patients with T1DM (10 studies, 226 patients, mean age 19.51 years) or T2DM (11 studies, 386 patients, mean age 52.91 years). These patients had an average diabetes history ranging from 0 (new-onset) to 15.8 years. BM-HSC (T1DM, 8 studies, 179 patients), MSC (T1DM, 2 studies, 47 patients; T2DM, 6 studies, 180 patients), and BM-MNC (T2DM, 6 studies, 216 patients) therapies were all included in this analysis. Among them, one study compared the efficacy of BM-MSCs and BM-MNCs in T2DM therapy [23]. Only nine articles included a control group, which received either insulin or placebo, with the experimental group receiving stem cell therapy. The follow-up period ranged from 12 weeks to 48 months. Some of the research data were not included in various studies, such as body mass index, cholesterol and waist circumference, and these data are thus not shown.

Quality assessment scores ranged from 20 to 25 (maximum possible score of 32), with the average score being 21.7 (see Supplementary Table 2). The included studies all met the methodological quality criteria items of more than 60%. The most common weaknesses in the study methods were a lack of blinding, non-randomized design, insufficient information on compliance, presence of confounding factors, and lack of information for determining whether the experimental population was representative.

**Figure 2.**
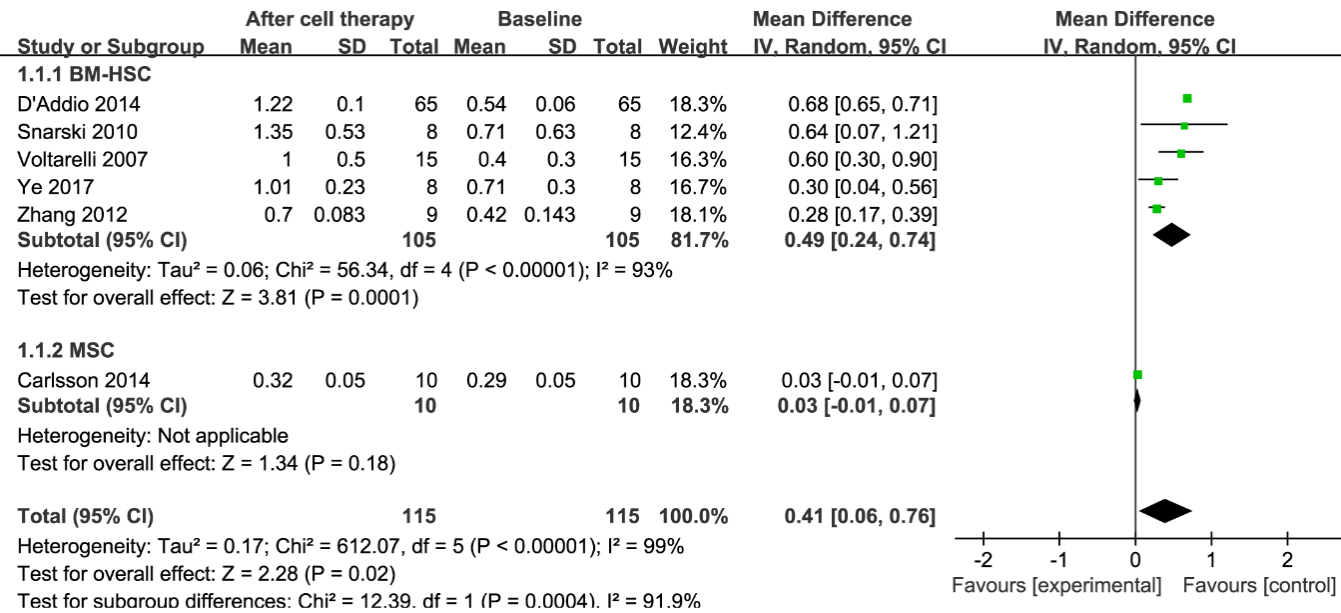
**Forest plot of C-peptide levels in type 1 diabetes mellitus (T1DM)**. Comparison of C-peptide levels in T1DM individuals between baseline and 12 months after stem cell therapy. A random-effects meta-analysis model (Mantel-Haenszel method) was used in this analysis. Each trial is represented by a square, the center of which denotes the mean difference (MD) for that trial. The size of the square is proportional to the information in that trial. The ends of the horizontal bars denote a 95% confidence interval. The black diamond gives the overall MD for all trials combined.

### Outcome of stem cell therapy for T1DM

Ten studies (226 patients, including 33 controls) were included in the analysis of stem cell therapy for T1DM.

### C-peptide

C-peptide levels reflect the level of synthesis of endogenous insulin, even in individuals receiving exogenous insulin. Information on C-peptide was available in six trials, including 115 patients treated with stem cell therapy [16, 18, 24-27]. The estimated pooled MD for these six trials revealed a significant increase in C-peptide at the 12-month follow-up (MD, 0.41; 95% CI, 0.06 to 0.76; *P < 0.001*). Regarding the effects of BM-HSC therapy, at the 12-month follow-up there was a significant increase in the estimated pooled MD in C-peptide for the five trials with 105 patients (MD, 0.49; 95% CI, 0.24 to 0.74; *P < 0.001*) [16, 18, 24-26]. In contrast, there was no significant change in an MSC therapy group with 10 patients (MD, 0.03; 95% CI, -0.01 to 0.07) [27]; however, only one study with C-peptide data included an MSC therapy group. These results are shown in Figure 2.

The pooled analysis showed significant heterogeneity in C-peptide levels. Sensitivity analysis revealed that one study [18] had a major impact on this heterogeneity (see Supplementary Table 3A). After excluding that study, the heterogeneity decreased significantly.

### HbA1c

**Figure 3.**
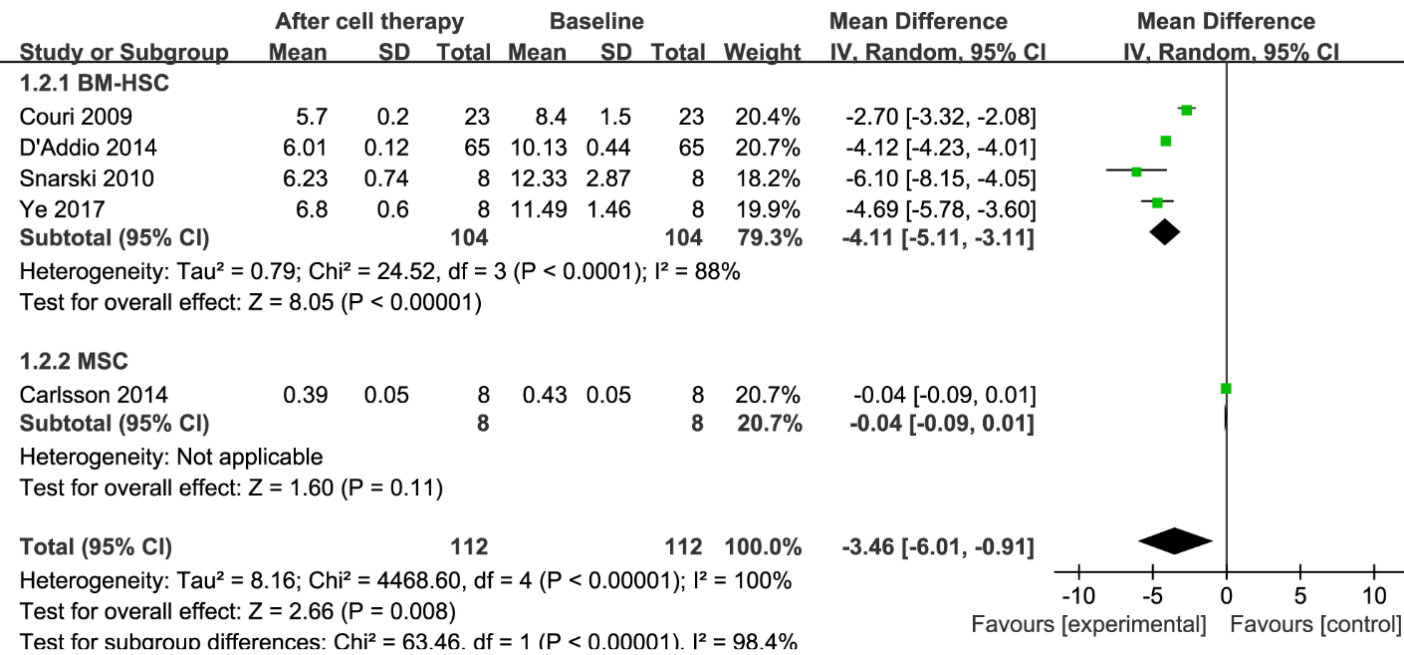
**Forest plot for glycosylated hemoglobin (HbA1c) in T1DM**. Comparison of HbA1c levels in T1DM individuals between baseline and 12 months after stem cell therapy. A random-effects meta-analysis model (Mantel-Haenszel method) was used in this analysis.

HbA1c is a stable marker that primarily reflects the average FPG level in the past 3 months. Information on HbA1c was available in five trials including 112 patients receiving stem cell therapy [18, 24, 25, 27, 28]. At the 12-month follow-up, stem cell therapy resulted in a significant decrease in HbA1c, as reflected in the estimated pooled MD of HbA1c for these five trials (MD, -3.46; 95% CI, -6.01 to -0.91; *P < 0.001*). Regarding the efficacy of BM-HSC therapy, at 12 months the estimated pooled MD of HbA1c for the four trials with 104 patients showed a significant decrease (MD,-4.11; 95% CI, -5.11 to -3.11; *P < 0.001*) [18, 24, 25, 28]. However, in the MSC therapy group, there was no significant improvement in one trial with eight patients [27] (MD, -0.04; 95% CI, -0.09 to 0.01). These results are shown in Figure 3.

The pooled analysis showed significant heterogeneity in HbA1c levels. Sensitivity analysis showed that one study [28] had a major impact on this heterogeneity (see Supplementary Table 3B). After excluding that study, the heterogeneity decreased significantly.

**Table 1:** Characteristics of the included studies.

Author and year	Country	Sample size (cell therapy/ control)	Male (%) (cell therapy/control)	Mean age (cell therapy/control) (years)	History of DM	Regimen	Regimens (cell number) dose	Injection mode	Mean follow-up period
Ye 2017 [25]	China	8/10(T1DM)	37.5% /40%	18.86 /20.18	<6 m	BM-HSC	NA	IV	12 m
D'Addio 2014 [18]	Poland	65(T1DM)	63%	20.4	<12 m	BM-HSC	5.8 ×l0^6^ /kg	IV	48 m
Zhang 2012 [26]	China	9(T1DM)	55.6%	17.6	2 y	BM-HSC	12.31 ×l0^6^ /kg	IV	12 m
Li 2012 [39]	China	13(T1DM)	69.2%	14.1	<12 m	BM-HSC	4 ×l0^6^ /kg	IV	42 m
Gu 2012 [44]	China	28(T1DM)	50%	17.6	3 m	BM-HSC	NA	IV	19.3 m
Snarski 2010 [24]	Poland	8(T1DM)	50%	25.8	2 m	BM-HSC	4.14×l0^6^ /kg	IV	7 m
Couri 2009 [28]	Brazil	23(T1DM)	73.9%	18.4	<2 m	BM-HSC	10.52×l0^6^ /kg	IV	29.8 m
Voltarelli 2007 [16]	Brazil	15(T1DM)	73.3%	19.2	<2 m	BM-HSC	11 ×l0^6^ /kg	IV	18.8 m
Carlsson 2014 [27]	Sweden	9/9(T1DM)	88.9% /55.6%	24 /27	<3 w	MSC	2.75 ×l0^6^ /kg	IV	12 m
Hu 2013 [36]	China	15/14(T1DM)	60% /57.1%	17.6 /18.2	New onset	MSC	2.6 ×10^7^/kg	IV	21 m
Bhansali 2017 [23]	India	10/10(T2DM)	80% /60%	50.5 /53.5	14.5 y	MSC	1 ×l0^6^ /kg	Superior pancreatico-duodenal artery	12 m
Hu 2016 [34]	China	31/30(T2DM)	54.8% /53.3%	52.43 /53.21	8.95 /8.3 y	MSC	6.1 ×10^7^	IV	36 m
Skyler 2015 [47]	USA	45/16(T2DM)	62.2% /75%	56.7 /58.7	10.1y	MSC	1.1×10^6^/kg	IV	12 w
Guan 2015 [48]	China	6(T2DM)	100%	40.5	42.7 w	MSC	0.88×10^6^/kg	IV	33.2 m
Liu 2014 [30]	China	22(T2DM)	68.18%	52.9	8.7 y	MSC	1×10^6^/kg	IV on Day 5+ Splenic artery on Day 10	12 m
Jiang 2011 [29]	China	10(T2DM)	70%	66	11 y	MSC	1.35 ×10^6^	IV	6 m
Bhansali 2017 [33]	India	7(T2DM)	85.7%	46	15 y	BM-MNC	1.2 ×10^9^	Superior pancreatico-duodenal artery	6 m
Bhansali 2017 [23]	India	10/10(T2DM)	70% /60%	44.5 /53.5	13.5 y	BM-MNC	1 ×10^9^	Superior pancreatico-duodenal artery	12 m
Wu 2014 [32]	China	20/20(T2DM)	60% /55.5%	56.4 /54.9	9.7 y	BM-MNC	4.01×10^9^	Dorsal pancreatic artery	12 m
Bhansali 2014 [31]	India	11/10(T2DM)	81.8% /70%	51 /54	15.8 y	BM-MNC	2.9 ×10^8^	Superior pancreatico-duodenal artery	12 m
Hu 2012 [35]	China	56/62(T2DM)	67.8% /58%	50.4 /50.2	8.6 y	BM-MNC	2.8 ×10^9^	Dorsal pancreatic artery	33 m
Bhansali 2009 [17]	India	10(T2DM)	80%	57.5	14.6 y	BM-MNC	3.5 × 10^8^	Superior pancreatico-duodenal artery	6 m

Abbreviations: DM: diabetes mellitus; T1DM: type 1 diabetes mellitus; T2DM: type 2 diabetes mellitus; BM-HSCs: bone marrow hematopoietic stem cells; MSCs: mesenchymal stem cells; BM-MNCs: bone marrow mononuclear cells; NA: not available; IV: intravenous.

### Insulin requirement

Because the data on insulin requirement were not always sufficient, we did not include these data in the meta-analysis. After BM-HSC transplantation, exogenous insulin requirements decreased significantly during the follow-up period, and 91 of 152 (59.9%) patients achieved exogenous insulin independence. In MSC transplantation therapy, insulin independence was observed in 3 of 15 patients (20%) over a mean period of 21 months, and in 8 patients (53.3%) the daily insulin dose was reduced by more than 50% relative to baseline. Overall, a gradual decrease in the exogenous insulin requirement was observed after stem cell therapy.

### Adverse events

Among the 24 patients who underwent MSC transplantation, there were no obvious adverse reactions to stem cell infusion or administration, such as mortality, tumor, or chronic infection. Of the 169 patients who underwent HSC transplantation, most experienced mild side effects only, such as nausea, vomiting, fever, or alopecia. However, 10 patients (5.9%) reported infection, 3 (1.8%) had endocrine dysfunction (hypothyroidism or hypogonadism), and 1 (0.59%) died.

### Outcome of stem cell therapy for T2DM

Eleven studies (386 patients, including 148 controls) were included in the analysis of stem cell therapy for T2DM.

### C-peptide

**Figure 4.**
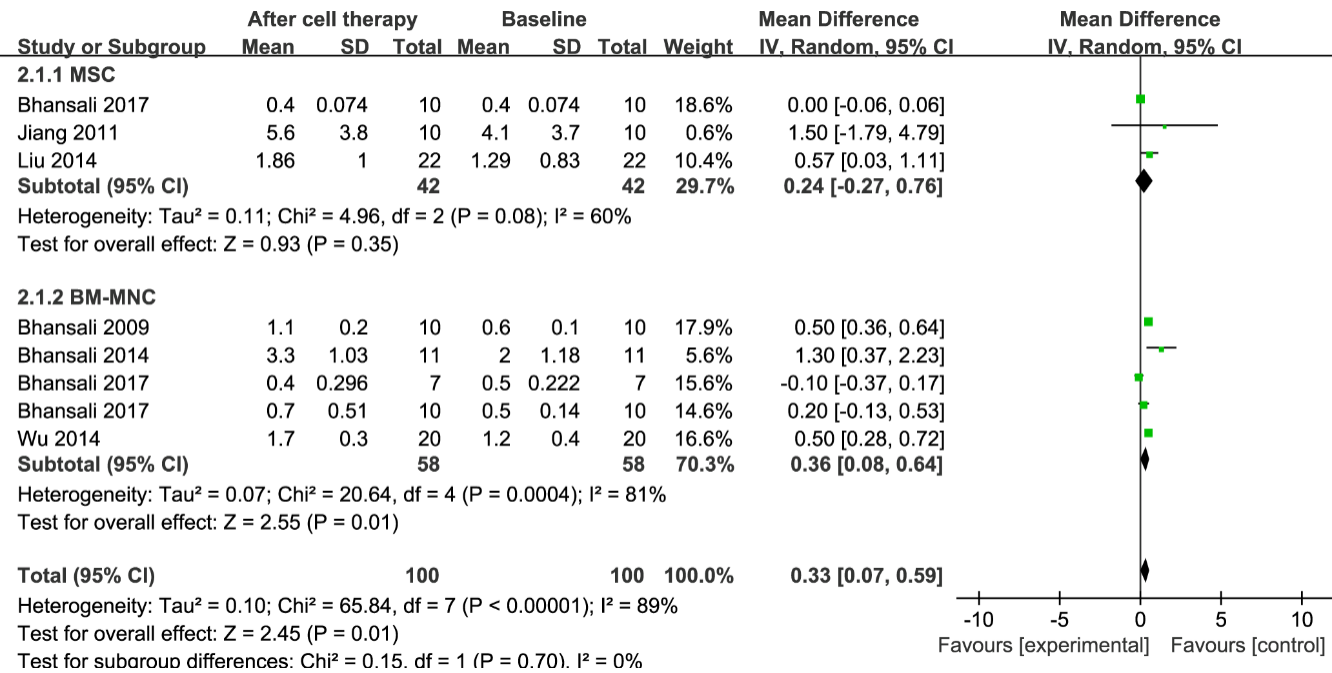
**Forest plot of C-peptide levels in type 2 diabetes mellitus (T2DM)**. Comparison of C-peptide levels in T2DM individuals between baseline and 12 months after stem cell therapy. A random-effects meta-analysis model (Mantel-Haenszel method) was used in this analysis.

Information on C-peptide was available in seven trials including 100 patients receiving stem cell therapy [17, 23, 29-33]. The estimated pooled MD for these seven trials revealed a significant increase in C-peptide (MD, 0.33; 95% CI, 0.07 to 0.59; *P < 0.001*). There was no change in C-peptide in the MSC group of three trials including 42 patients (MD, 0.24; 95% CI, -0.27 to 0.76; *P = 0.08*) [23, 29, 30]. Regarding the efficacy of BM-MNC therapy at 12 months, there was a significant increase in the estimated pooled MD for C-peptide for five trials including 58 patients (MD, 0.36; 95% CI, 0.08 to 0.64; *P < 0.001*) [17, 23, 31-33]. These results are shown in Figure 4.

The pooled analysis showed significant heterogeneity in C-peptide levels. In the MSC treatment group, sensitivity analysis showed that two studies [23, 30] had a major effect on this heterogeneity (see Supplementary Table 4A). When either of these two studies was removed, the heterogeneity was significantly reduced. In the BM-MNC treatment group, sensitivity analysis showed that one study [33] had a major effect on the heterogeneity (see Supplementary Table 4B). After that study had been excluded, the heterogeneity decreased significantly.

### HbA1c

**Figure 5.**
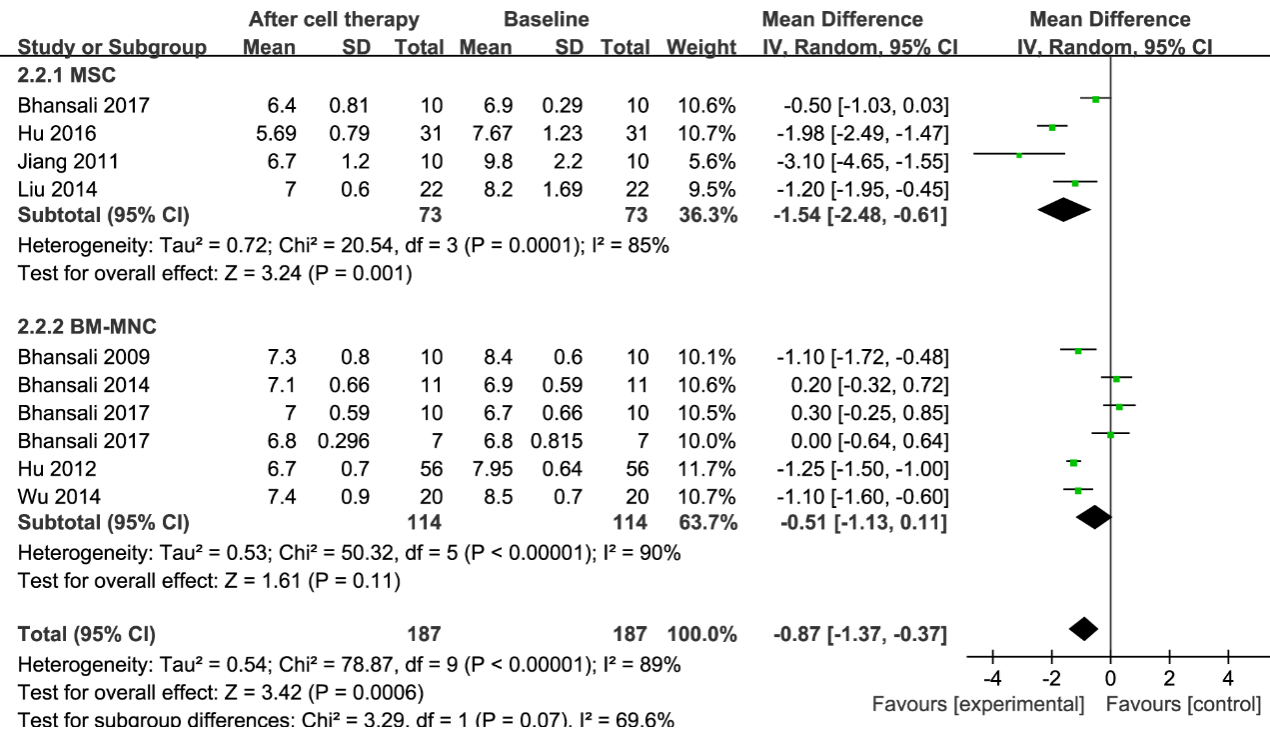
**Forest plot of HbA1c in T2DM**. Comparison of HbA1c levels in T2DM individuals between baseline and 12 months after stem cell therapy. A random-effects meta-analysis model (Mantel-Haenszel method) was used in this analysis.

Information on HbA1c was available in nine trials, including 187 patients receiving stem cell therapy [17, 23, 29-35]. The estimated pooled MD in HbA1c for those nine trials showed a significant reduction after stem cell therapy (MD, -0.87; 95% CI, -1.37 to -0.37; *P < 0.001*). A significant reduction in HbA1c was observed in the MSC group (73 patients in four studies; MD, -1.54; 95% CI, -2.48 to -0.61; *P < 0.001*) [23, 29, 30, 34]. Regarding the efficacy of BM-MNC after 12 months of therapy, the estimated pooled MD of HbA1c for six trials including 114 patients did not show a significant improvement (MD, -0.51; 95% CI, -1.13 to 0.11; *P < 0.001*) [17, 23, 31-33, 35]. These results are shown in Figure 5.

The pooled analysis showed significant heterogeneity in HbA1c levels. In the MSC treatment group, sensitivity analysis showed that one study [23] had a major effect on the heterogeneity (see Supplementary Table 4C). After that study was excluded, the heterogeneity decreased significantly. In the BM-MNC treatment group, regardless of the removal of any study, the heterogeneity was not significantly reduced (see Supplementary Table 4D).

### Insulin requirements

**Figure 6.**
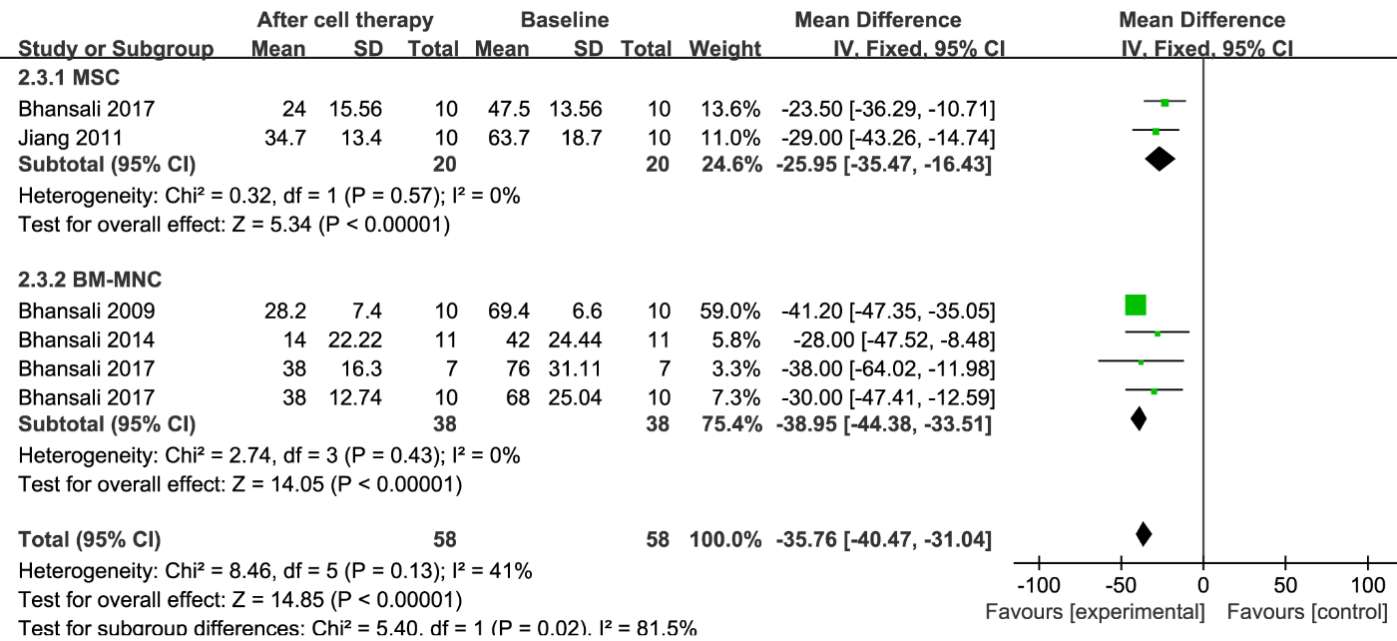
**Forest plot of insulin requirement in T2DM**. Comparison of insulin requirement in T2DM individuals between baseline and 12 months after stem cell therapy. A fixed-effects meta-analysis model (Mantel-Haenszel method) was used in this analysis.

Information on insulin requirement was available in five trials that included 58 patients receiving stem cell therapy [17, 23, 29, 31, 33]. The estimated pooled MD for these five trials showed a significant reduction in exogenous insulin requirement at the 12-month follow-up (MD, -35.76; 95% CI, -40.47 to -31.04; *P = 0.13*). Significant decreases in insulin requirements were observed in the MSC group (two trials including 20 patients) after 12 months of therapy (MD, -25.95; 95% CI, -35.47 to -16.43; *P = 0.57*) [23, 29]. Similarly, regarding the efficacy of BM-MNC therapy, exogenous insulin requirement at 12 months showed a significant reduction, as reflected by the estimated pooled MD for four trials including 31 patients (MD, -38.95; 95% CI, -44.38 to -33.51; *P = 0.43*) [17, 23, 31, 33]. Exogenous insulin was discontinued in 20 of 64 (31.25%) patients after MSC transplantation, and 27 of 64 (42.2%) patients showed a more than 50% reduction in insulin requirement. Of the 94 patients who received BM-MNC transplantation, exogenous insulin was discontinued in 20 patients (21.3%), the daily insulin dosage was reduced by more than 50% of the baseline in 44 patients (46.8%), and the daily insulin dosage was reduced by 15-50% in 18 patients (19.1%). These results are shown in Figure 6.

### FPG

**Figure 7.**
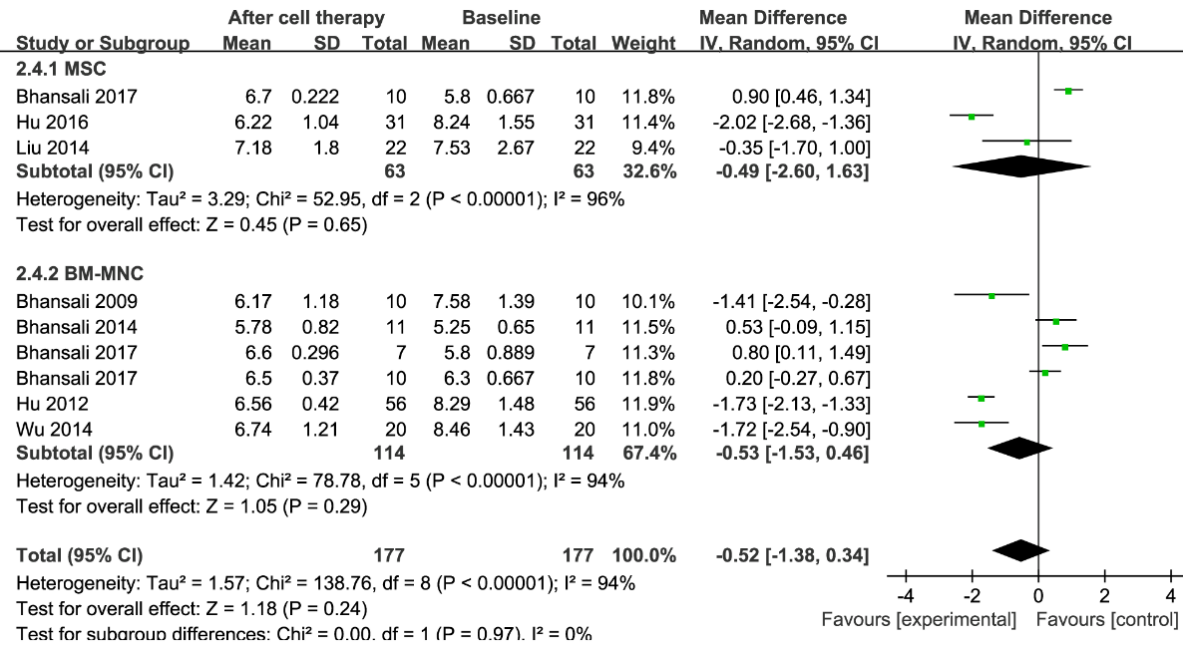
**Forest plot of fasting plasma glucose (FPG) in T2DM**. Comparison of FPG levels in T2DM individuals between baseline and 12 months after stem cell therapy. A random-effects meta-analysis model (Mantel-Haenszel method) was used in this analysis.

Information regarding FPG was available in eight trials that included 177 patients receiving stem cell therapy [17, 23, 30-35]. The estimated pooled MD for the eight trials showed no significant improvement in FPG levels (MD, -0.52; 95% CI, -1.38 to 0.34;* P < 0.001*). There was no obvious change in FPG level in the MSC group for three trials including 63 patients (MD, -0.49; 95% CI, -2.60 to 1.63; *P < 0.001*) [23, 30, 34]. Regarding the efficacy of BM-MNC therapy at the 12-month follow-up, no significant reduction in the estimated pooled MD was seen for six trials including 114 patients (MD, -0.53; 95% CI, -1.53 to 0.46; *P < 0.001*) [17, 23, 31-33, 35]. These results are shown in Figure 7.

The pooled analysis showed significant heterogeneity in FPG levels. In the MSC treatment group, a sensitivity analysis showed that one study [34] had a major effect on the heterogeneity (see Supplementary Table 4E). After that study was excluded, the heterogeneity decreased significantly. In the BM-MNC treatment group, regardless of which study was removed, the heterogeneity was not significantly reduced (see Supplementary Table 4F).

### Adverse events

The side effects of MSC transplantation included mild and moderate fever in 3 of the 124 patients (2.42%) and nausea, vomiting, and headache in 1 patient (0.81%). Of the 114 patients receiving BM-MNC transplantation, nausea and vomiting occurred in 7 patients (6.14%), injection site hematoma developed in 1 patient (0.88%), punctate hemorrhage occurred in 3 patients (2.63%), and abdominal pain occurred in 3 patients (2.63%). These stem cell therapy-related adverse reactions were mild, and patients recovered spontaneously.

## DISCUSSION

This meta-analysis evaluated the clinical efficacy and safety of stem cell transplantation for patients with T1DM and T2DM in several countries. We demonstrated that stem cell therapy could improve the levels of C-peptide, HbA1c, and insulin requirements for T1DM patients.

To determine the impact of different stem cells on diabetes treatment, we conducted subgroup meta-analyses according to cell type. Our analysis showed significant HbA1c reduction and C-peptide improvement in patients with T1DM treated with BM-HSC. Additionally, 59.9% of treated T1DM patients achieved exogenous insulin independence after BM-HSC transplantation therapy. However, there was no significant reduction in HbA1c or improvement in C-peptide levels in T1DM patients who received MSC treatment, and only 20% of treated T1DM patients achieved exogenous insulin independence. In particular, MSCs can be isolated from a variety of tissues including bone marrow, umbilical cord, placenta, and adipose tissues. In the studies we included, MSCs were derived from bone marrow or umbilical cord tissue. Carlsson et al. reported no significant difference in HbA1c, insulin requirements, or C-peptide levels after treatment with bone marrow-derived MSCs [27]. Hu et al. reported that both the HbA1c and C-peptide levels in umbilical cord-derived MSC group patients were significantly better than both pre-therapy values and those in control group patients during the follow-up period [36]. Therefore, umbilical cord-derived MSCs appear to be more effective than bone marrow-derived MSCs in the treatment of T1DM. However, there are few studies on MSC treatment for T1DM, and the sample size in this study was small. More studies are needed to explore which source of MSCs is most suitable for treatment. In conclusion, BM-HSCs showed better efficacy in improving C-peptide levels, HbA1c levels, and exogenous insulin requirements than did MSCs. A study in alloxan-induced diabetic rats also showed better differentiation capacity of BM-HSCs into insulin-producing cells (IPCs) than BM-MSCs [37]. In a diabetic model induced by streptozotocin, transplantation of both MSCs and HSCs derived from mouse bone marrow improved glycemic control in diabetic mice, but the course and mechanism may be different [38]. The mechanisms underlying the effect of stem cell transplantation in patients with T1DM are not yet fully understood. HSC transplantation improved islet cell function, possibly by eliminating islet-specific autoreactive T cells and reconstituting a decreased inflammatory environment [26, 39]. The efficacy of MSC treatment for T1DM may be due to its strong immune-regulating ability [40-43]. Randomized controlled trials and further studies with a large number of cases are warranted to verify the therapeutic effect of HSC transplantation and determine the mechanism of action of HSC transplantation in patients with T1DM.

In the majority of T1DM patients treated with stem cells, either no significant adverse reactions occurred or mild adverse reactions recovered spontaneously [28, 44]. However, with HSC treatment of T1DM, three individuals had serious infectious diseases, and one of them died of sepsis due to Pseudomonas aeruginosa infection [18]. Infectious diseases may arise due to the administration of high-dose immunosuppressive therapy to mobilize HSCs, although immune function is restored after autologous HSC transplantation [18]. Compared with other advanced autoimmune diseases, newly diagnosed T1DM patients show a lower frequency of severe acute complications after HSC treatment [45]. In addition, three patients developed endocrine dysfunction (hypothyroidism or hypogonadism) [16, 39], which may have been caused by autoimmune disorders associated with transplant surgery [46]. The occurrence of severe adverse events in treated subjects also highlighted the fact that HSC transplantation may represent a potential therapeutic approach for selected individuals with T1DM but is most likely not suitable for all patients. Moreover, this finding also confirms that further studies on safer treatment options based on stem cells are needed.

Our study also showed that stem cell therapy could improve the levels of C-peptide and HbA1c, and insulin requirements, for T2DM patients. However, there was no significant change in FPG levels.

We also conducted a subgroup meta-analysis of the clinical efficacy of stem cell transplantation for the treatment of T2DM. We found that HbA1c and daily insulin requirements decreased significantly after MSC transplantation in patients with T2DM. However, there was no significant improvement in C-peptide or FPG levels after MSC transplantation. Our analysis showed that after treatment with BM-MNC in patients with T2DM, insulin requirements and C-peptide levels were significantly improved, while HbA1c and FPG levels did not change significantly. Moreover, Wu et al. showed a significant increase in quality of life (Short-Form Health Survey; SF-36) score during follow-up after BM-MNC treatment [32]. Hu et al. found that homeostasis model assessment of pancreatic islet β-cell function in an MSC infusion treatment group significantly increased during the follow-up period compared with baseline levels [34]. Similarly, a comparative study of BM-MSCs and BM-MNCs for the treatment of T2DM showed a significant increase in insulin sensitivity in the MSC-treated group, while BM-MNC increased the C-peptide response; however, both BM-MSC and BM-MNC transplantation can lead to a decrease in the dose of exogenous insulin in patients with T2DM [23]. In the studies we included, MSCs were derived from bone marrow, placenta, or umbilical cord tissue. Both Skyler et al. and Bhansali et al. have shown that patients with T2DM have reduced exogenous insulin requirements after bone marrow-derived MSC treatment [23, 47]. Jiang et al. found that placenta-derived stem cell therapy improves C-peptide, HbA1c levels, and insulin requirements, in T2DM patients [29]. Similarly, umbilical cord-derived MSC therapy can significantly improve C-peptide levels and insulin requirements in patients with T2DM [30, 34, 48]. Bone marrow, placenta and umbilical cord-derived MSCs can improve islet function in T2DM, but it is unclear which source of MSC is most suitable for treating the disease. To our knowledge, there are no reports comparing different sources of MSCs for the treatment of T2DM. Moreover, the number of studies available is too small to enable subgroup meta-analysis of MSCs derived from different sources.

The above results suggest that stem cell infusion could enhance the function of islet β-cells in T2DM patients. Although the mechanism of action of stem cell therapy for T2DM remains unclear, the mechanism underlying the improvement in β-cell function may involve the following: (1) stem cells can differentiate into islet cells and then secrete insulin [49-51]; (2) stem cells differentiate into vascular endothelial cells, which can improve blood supply to the pancreas and restore β-cell function [52]; (3) MSC transplantation increases glucose transporter 4 expression, and levels of phosphorylated insulin receptor substrate 1 and Akt (protein kinase B) in insulin target tissues [53]; and (4) a relatively small quantity of transplanted MSC can transdifferentiate into IPCs in the pancreas [54]. In addition, studies have shown that MSCs can produce high levels of anti-apoptotic signaling molecules to improve the pancreatic microenvironment and enhance the expansion of endogenous pancreatic stem cells [55, 56]. MSC transplantation also reduces the levels of pro-inflammatory cytokines such as interleukin (IL)-6 and IL-1β, which are involved in the development of insulin resistance [30, 57, 58].

In the patients with T2DM who received stem cell therapy, either no significant adverse reactions occurred, or mild adverse reactions spontaneously recovered. These results suggested that MSCs may be a safe therapeutic approach for patients with T2DM.

Substantial heterogeneity was observed among the included studies. The sensitivity analyses suggested that heterogeneity could be decreased by excluding only one study. However, two pooled analyses showed that heterogeneity remained high after omitting any study; thus, the results should be interpreted with caution. Heterogeneity might have arisen from the non-homogeneity in the baseline characteristics of the subjects, ethnic background, diet, lifestyle, number of stem cell transplants, or timing or route of stem cell transplantation. In addition, differences in follow-up time and the degree of progression of diabetes may also have been sources of heterogeneity among the included studies. Therefore, additional basic and clinical studies are needed to test assess possible mechanisms of stem cell transplantation for diabetes, and consensus is required regarding the criteria for stem cell transplantation.

Although our meta-analysis shows that stem cell transplantation is safe and effective for most people with diabetes, there were several limitations that must be considered. First, because some subgroups comprised patients from few studies, we evaluated a single study with lower statistical ability. Also, the total sample size was not very large, and the follow-up period was short. Furthermore, the methodologies of the included studies were generally poor. Due to ethical issues, many of these trials were not blinded or did not involve randomization, which may have resulted in large performance and measurement biases. To reduce measurement bias, we assessed the average change in patients with respect to baseline measurements, but this approach may have also affected the reliability of the results. Moreover, only nine articles included a control group, for which data on relevant laboratory diabetes parameters were not available. Thus, we made no comparison between control and experimental groups. Lastly, some information about the patients was not available, such as their diet and use of medications, which may have also affected the observed outcomes.

### Conclusion

In conclusion, the results of this meta-analysis showed improvements in C-peptide level, HbA1c level, and daily exogenous insulin requirement after stem cell treatment for diabetes. Stem cell therapy may be a safe and effective intervention for selected individuals with diabetes. In T1DM, BM-HSCs are a good source for stem cell transplantation. In T2DM, HbA1c and daily insulin requirements were significantly improved after MSC therapy, and BM-MNC therapy significantly improved insulin requirements and C-peptide levels. These encouraging results require validation in larger, randomized, double-blind studies, as well as longer follow-up periods to establish stem cell-based therapies as the standard of care for treating DM.

## Supplementary Materials

The Supplemenantry data can be found online at: www.aginganddisease.org/EN/10.14336/AD.2019.0421.

## References

[1] International Diabetes Federation.IDF Diabetes Atlas 8th edn. Brussels, Belgium: International Diabetes Federation, 2017.

[2] American Diabetes Association (2004). Diagnosis and classification of diabetes mellitus. Diabetes Care, 27 Suppl 1:S5-s10. 14693921 10.2337/diacare.27.2007.s5

[3] Kolb H, Mandrup-Poulsen T (2005). An immune origin of type 2 diabetes? Diabetologia, 48:1038-1050. 15864529 10.1007/s00125-005-1764-9

[4] Todd JA (2010). Etiology of type 1 diabetes. Immunity, 32:457-467. 20412756 10.1016/j.immuni.2010.04.001

[5] Melendez-Ramirez LY, Richards RJ, Cefalu WT (2010). Complications of type 1 diabetes. Endocrinol Metab Clin North Am, 39:625-640. 20723824 10.1016/j.ecl.2010.05.009

[6] Das Evcimen N, King G (2007). The role of protein kinase C activation and the vascular complications of diabetes. Pharmacol. Res., 55:498-510. 17574431 10.1016/j.phrs.2007.04.016

[7] Peng BY, Dubey NK, Mishra VK, Tsai FC, Dubey R, Deng WP, et al (2018). Addressing Stem Cell Therapeutic Approaches in Pathobiology of Diabetes and Its Complications. J Diabetes Res, 2018:7806435. 30046616 10.1155/2018/7806435 PMC6036791

[8] Lilly MA, Davis MF, Fabie JE, Terhune EB, Gallicano GI (2016). Current stem cell based therapies in diabetes. Am J Stem Cells, 5:87-98. 27853630 PMC5107653

[9] Teramura Y, Iwata H (2010). Bioartificial pancreas microencapsulation and conformal coating of islet of Langerhans. Adv Drug Deliv Rev, 62:827-840. 20138097 10.1016/j.addr.2010.01.005

[10] Gaba RC, Garcia-Roca R, Oberholzer J (2012). Pancreatic islet cell transplantation: an update for interventional radiologists. J Vasc Interv Radiol, 23:583-594; quiz 594. 22417970 10.1016/j.jvir.2012.01.057

[11] Robertson RP (2004). Islet transplantation as a treatment for diabetes - a work in progress. N Engl J Med, 350:694-705. 14960745 10.1056/NEJMra032425

[12] Gibly RF, Graham JG, Luo X, Lowe WL Jr, Hering BJ, Shea LD (2011). Advancing islet transplantation: from engraftment to the immune response. Diabetologia, 54:2494-2505. 21830149 10.1007/s00125-011-2243-0 PMC3193607

[13] Chhabra P, Brayman KL (2013). Stem cell therapy to cure type 1 diabetes: from hype to hope. Stem Cells Transl Med, 2:328-336. 23572052 10.5966/sctm.2012-0116 PMC3667565

[14] Hess D, Li L, Martin M, Sakano S, Hill D, Strutt B, et al (2003). Bone marrow-derived stem cells initiate pancreatic regeneration. Nat Biotechnol, 21:763-770. 12819790 10.1038/nbt841

[15] Pera MF, Tam PP (2010). Extrinsic regulation of pluripotent stem cells. Nature, 465:713-720. 20535200 10.1038/nature09228

[16] Voltarelli JC, Couri CEB, Stracieri ABPL, Oliveira MC, Moraes DA, Pieroni F, et al (2007). Autologous nonmyeloablative hematopoietic stem cell transplantation in newly diagnosed type 1 diabetes mellitus. Journal of the American Medical Association, 297:1568-1576. 17426276 10.1001/jama.297.14.1568

[17] Bhansali A, Upreti V, Khandelwal N, Marwaha N, Gupta V, Sachdeva N, et al (2009). Efficacy of autologous bone marrow-derived stem cell transplantation in patients with type 2 diabetes mellitus. Stem Cells Dev, 18:1407-1416. 19686048 10.1089/scd.2009.0164

[18] D'Addio F, Valderrama Vasquez A, Ben Nasr M, Franek E, Zhu D, Li L, et al (2014). Autologous nonmyeloablative hematopoietic stem cell transplantation in new-onset type 1 diabetes: a multicenter analysis. Diabetes, 63:3041-3046. 24947362 10.2337/db14-0295

[19] Rahim F, Arjmand B, Shirbandi K, Payab M, Larijani B (2018). Stem cell therapy for patients with diabetes: a systematic review and meta-analysis of metabolomics-based risks and benefits. Stem Cell Investig, 5:40. 10.21037/sci.2018.11.01 PMC6286886 30596080

[20] El-Badawy A, El-Badri N (2016). Clinical Efficacy of Stem Cell Therapy for Diabetes Mellitus: A Meta-Analysis. PLoS One, 11:e0151938. 27073927 10.1371/journal.pone.0151938 PMC4830527

[21] Moher D, Liberati A, Tetzlaff J, Altman DG (2009). Preferred reporting items for systematic reviews and meta-analyses: the PRISMA statement. J Clin Epidemiol, 62:1006-1012. 19631508 10.1016/j.jclinepi.2009.06.005

[22] Downs SH, Black N (1998). The feasibility of creating a checklist for the assessment of the methodological quality both of randomised and non-randomised studies of health care interventions. J Epidemiol Community Health, 52:377-384. 9764259 10.1136/jech.52.6.377 PMC1756728

[23] Bhansali S, Dutta P, Kumar V, Yadav MK, Jain A, Mudaliar S, et al (2017). Efficacy of Autologous Bone Marrow-Derived Mesenchymal Stem Cell and Mononuclear Cell Transplantation in Type 2 Diabetes Mellitus: A Randomized, Placebo-Controlled Comparative Study. Stem Cells Dev, 26:471-481. 28006991 10.1089/scd.2016.0275

[24] Snarski E, Milczarczyk A, Torosian T, Paluszewska M, Urbanowska E, Krol M, et al (2011). Independence of exogenous insulin following immunoablation and stem cell reconstitution in newly diagnosed diabetes type I. Bone Marrow Transplant, 46:562-566. 20581881 10.1038/bmt.2010.147

[25] Ye L, Li L, Wan B, Yang M, Hong J, Gu W, et al (2017). Immune response after autologous hematopoietic stem cell transplantation in type 1 diabetes mellitus. Stem Cell Res Ther, 8:90. 28420440 10.1186/s13287-017-0542-1 PMC5395765

[26] Zhang X, Ye L, Hu J, Tang W, Liu R, Yang M, et al (2012). Acute response of peripheral blood cell to autologous hematopoietic stem cell transplantation in type 1 diabetic patient. PLoS One, 7:e31887. 22384093 10.1371/journal.pone.0031887 PMC3285188

[27] Carlsson PO, Schwarcz E, Korsgren O, Le Blanc K (2015). Preserved beta-cell function in type 1 diabetes by mesenchymal stromal cells. Diabetes, 64:587-592. 25204974 10.2337/db14-0656

[28] Couri CE, Oliveira MC, Stracieri AB, Moraes DA, Pieroni F, Barros GM, et al (2009). C-peptide levels and insulin independence following autologous nonmyeloablative hematopoietic stem cell transplantation in newly diagnosed type 1 diabetes mellitus. Jama, 301:1573-1579. 19366777 10.1001/jama.2009.470

[29] Jiang R, Han Z, Zhuo G, Qu X, Li X, Wang X, et al (2011). Transplantation of placenta-derived mesenchymal stem cells in type 2 diabetes: a pilot study. Front Med, 5:94-100. 21681681 10.1007/s11684-011-0116-z

[30] Liu X, Zheng P, Wang X, Dai G, Cheng H, Zhang Z, et al (2014). A preliminary evaluation of efficacy and safety of Wharton's jelly mesenchymal stem cell transplantation in patients with type 2 diabetes mellitus. Stem Cell Res Ther, 5:57. 24759263 10.1186/scrt446 PMC4055092

[31] Bhansali A, Asokumar P, Walia R, Bhansali S, Gupta V, Jain A, et al (2014). Efficacy and safety of autologous bone marrow-derived stem cell transplantation in patients with type 2 diabetes mellitus: a randomized placebo-controlled study. Cell Transplant, 23:1075-1085. 23561959 10.3727/096368913X665576

[32] Wu Z, Cai J, Chen J, Huang L, Wu W, Luo F, et al (2014). Autologous bone marrow mononuclear cell infusion and hyperbaric oxygen therapy in type 2 diabetes mellitus: an open-label, randomized controlled clinical trial. Cytotherapy, 16:258-265. 24290656 10.1016/j.jcyt.2013.10.004

[33] Bhansali S, Dutta P, Yadav MK, Jain A, Mudaliar S, Hawkins M, et al (2017). Autologous bone marrow-derived mononuclear cells transplantation in type 2 diabetes mellitus: effect on beta-cell function and insulin sensitivity. Diabetol Metab Syndr, 9:50. 28690682 10.1186/s13098-017-0248-7 PMC5496640

[34] Hu J, Wang Y, Gong H, Yu C, Guo C, Wang F, et al (2016). Long term effect and safety of Wharton's jelly-derived mesenchymal stem cells on type 2 diabetes. Exp Ther Med, 12:1857-1866. 27588104 10.3892/etm.2016.3544 PMC4997981

[35] Hu J, Li C, Wang L, Zhang X, Zhang M, Gao H, et al (2012). Long term effects of the implantation of autologous bone marrow mononuclear cells for type 2 diabetes mellitus. Endocr J, 59:1031-1039. 22814142 10.1507/endocrj.ej12-0092

[36] Hu J, Yu X, Wang Z, Wang F, Wang L, Gao H, et al (2013). Long term effects of the implantation of Wharton's jelly-derived mesenchymal stem cells from the umbilical cord for newly-onset type 1 diabetes mellitus. Endocr J, 60:347-357. 23154532 10.1507/endocrj.ej12-0343

[37] Azab NI, AlKholy AF, Salem RF, Gabr H, ElAbd AM (2011). Comparison between bone marrow derived mesenchymal stem cells and hematopoietic stem cells in β-Islet transdifferentiation. Stem Cells:2(1):1-10.

[38] Arany EJ, Waseem M, Strutt BJ, Chamson-Reig A, Bernardo A, Eng E, et al (2018). Direct comparison of the abilities of bone marrow mesenchymal versus hematopoietic stem cells to reverse hyperglycemia in diabetic NOD.SCID mice. Islets, 10:137-150. 30110202 10.1080/19382014.2018.1480285 PMC6281365

[39] Li L, Shen S, Ouyang J, Hu Y, Hu L, Cui W, et al (2012). Autologous hematopoietic stem cell transplantation modulates immunocompetent cells and improves β-cell function in Chinese patients with new onset of type 1 diabetes. Journal of Clinical Endocrinology and Metabolism, 97:1729-1736. 22419704 10.1210/jc.2011-2188

[40] English K, Ryan JM, Tobin L, Murphy MJ, Barry FP, Mahon BP (2009). Cell contact, prostaglandin E(2) and transforming growth factor beta 1 play non-redundant roles in human mesenchymal stem cell induction of CD4+CD25(High) forkhead box P3+ regulatory T cells. Clin Exp Immunol, 156:149-160. 19210524 10.1111/j.1365-2249.2009.03874.x PMC2673753

[41] Thakkar UG, Trivedi HL, Vanikar AV, Dave SD (2015). Insulin-secreting adipose-derived mesenchymal stromal cells with bone marrow-derived hematopoietic stem cells from autologous and allogenic sources for type 1 diabetes mellitus. Cytotherapy, 17:940-947. 25869301 10.1016/j.jcyt.2015.03.608

[42] Volarevic V, Al-Qahtani A, Arsenijevic N, Pajovic S, Lukic ML (2010). Interleukin-1 receptor antagonist (IL-1Ra) and IL-1Ra producing mesenchymal stem cells as modulators of diabetogenesis. Autoimmunity, 43:255-263. 19845478 10.3109/08916930903305641

[43] Abdi R, Fiorina P, Adra CN, Atkinson M, Sayegh MH (2008). Immunomodulation by mesenchymal stem cells: a potential therapeutic strategy for type 1 diabetes. Diabetes, 57:1759-1767. 18586907 10.2337/db08-0180 PMC2453631

[44] Gu W, Hu J, Wang W, Li L, Tang W, Sun S, et al (2012). Diabetic ketoacidosis at diagnosis influences complete remission after treatment with hematopoietic stem cell transplantation in adolescents with type 1 diabetes. Diabetes Care, 35:1413-1419. 22723579 10.2337/dc11-2161 PMC3379609

[45] Burt RK, Slavin S, Burns WH, Marmont AM (2002). Induction of tolerance in autoimmune diseases by hematopoietic stem cell transplantation: getting closer to a cure? Blood, 99:768-784. 11806976 10.1182/blood.v99.3.768

[46] Au WY, Lie AK, Kung AW, Liang R, Hawkins BR, Kwong YL (2005). Autoimmune thyroid dysfunction after hematopoietic stem cell transplantation. Bone Marrow Transplant, 35:383-388. 15640829 10.1038/sj.bmt.1704766

[47] Skyler JS, Fonseca VA, Segal KR, Rosenstock J (2015). Allogeneic Mesenchymal Precursor Cells in Type 2 Diabetes: A Randomized, Placebo-Controlled, Dose-Escalation Safety and Tolerability Pilot Study. Diabetes Care, 38:1742-1749. 26153271 10.2337/dc14-2830 PMC4542273

[48] Guan LX, Guan H, Li HB, Ren CA, Liu L, Chu JJ, et al (2015). Therapeutic efficacy of umbilical cord-derived mesenchymal stem cells in patients with type 2 diabetes. Exp Ther Med, 9:1623-1630. 26136869 10.3892/etm.2015.2339 PMC4471780

[49] Phadnis SM, Joglekar MV, Dalvi MP, Muthyala S, Nair PD, Ghaskadbi SM, et al (2011). Human bone marrow-derived mesenchymal cells differentiate and mature into endocrine pancreatic lineage in vivo. Cytotherapy, 13:279-293. 21039304 10.3109/14653249.2010.523108

[50] Carlotti F, Zaldumbide A, Loomans CJ, van Rossenberg E, Engelse M, de Koning EJ, et al (2010). Isolated human islets contain a distinct population of mesenchymal stem cells. Islets, 2:164-173. 21099310 10.4161/isl.2.3.11449

[51] Phuc PV, Nhung TH, Loan DT, Chung DC, Ngoc PK (2011). Differentiating of banked human umbilical cord blood-derived mesenchymal stem cells into insulin-secreting cells. In Vitro Cell Dev Biol Anim, 47:54-63. 21082287 10.1007/s11626-010-9356-5

[52] Liao W, Xie J, Zhong J, Liu Y, Du L, Zhou B, et al (2009). Therapeutic effect of human umbilical cord multipotent mesenchymal stromal cells in a rat model of stroke. Transplantation, 87:350-359. 19202439 10.1097/TP.0b013e318195742e

[53] Si Y, Zhao Y, Hao H, Liu J, Guo Y, Mu Y, et al (2012). Infusion of mesenchymal stem cells ameliorates hyperglycemia in type 2 diabetic rats: identification of a novel role in improving insulin sensitivity. Diabetes, 61:1616-1625. 22618776 10.2337/db11-1141 PMC3357293

[54] Dong QY, Chen L, Gao GQ, Wang L, Song J, Chen B, et al (2008). Allogeneic diabetic mesenchymal stem cells transplantation in streptozotocin-induced diabetic rat. Clin Invest Med, 31:E328-337. 19032902 10.25011/cim.v31i6.4918

[55] Park KS, Kim YS, Kim JH, Choi B, Kim SH, Tan AH, et al (2010). Trophic molecules derived from human mesenchymal stem cells enhance survival, function, and angiogenesis of isolated islets after transplantation. Transplantation, 89:509-517. 20125064 10.1097/TP.0b013e3181c7dc99

[56] Lee RH, Seo MJ, Reger RL, Spees JL, Pulin AA, Olson SD, et al (2006). Multipotent stromal cells from human marrow home to and promote repair of pancreatic islets and renal glomeruli in diabetic NOD/scid mice. Proc Natl Acad Sci U S A, 103:17438-17443. 17088535 10.1073/pnas.0608249103 PMC1634835

[57] Welsh N, Cnop M, Kharroubi I, Bugliani M, Lupi R, Marchetti P, et al (2005). Is there a role for locally produced interleukin-1 in the deleterious effects of high glucose or the type 2 diabetes milieu to human pancreatic islets? Diabetes, 54:3238-3244. 16249450 10.2337/diabetes.54.11.3238

[58] Kern PA, Ranganathan S, Li C, Wood L, Ranganathan G (2001). Adipose tissue tumor necrosis factor and interleukin-6 expression in human obesity and insulin resistance. Am J Physiol Endocrinol Metab, 280:E745-751. 11287357 10.1152/ajpendo.2001.280.5.E745

