# Supplementary material for: The Clinical Efficacy and Safety of Stem Cell Therapy for Diabetes Mellitus: A Systematic Review and Meta-Analysis
Source: Aging Dis. 2020 Feb 1;11(1):141–53. doi: 10.14336/AD.2019.0421 (PMC6961772; doi:10.14336/AD.2019.0421)
Supplement: Supplementary file 1 — The Supplemenantry data can be found online at: www.aginganddisease.org/EN/10.14336/AD.2019.0421. [file AD-11-1-141-s.pdf]

SUPPLEMENTARY DATA

The Clinical Efficacy and Safety of Stem Cell Therapy for Diabetes Mellitus: A Systematic Review and Meta-Analysis

Yazhen Zhang[1,2]， Wenyi Chen[1,2], Bing Feng[1,2], Hongcui Cao[1,2]*

[1]State Key Laboratory for Diagnosis and Treatment of Infectious Diseases, the First Affiliated Hospital, College of Medicine, Zhejiang University, Hangzhou, China
[2]Collaborative Innovation Center for Diagnosis and Treatment of Infectious Diseases, Hangzhou, China

© 2019. Zhang Y et al. Published online at http://www.aginganddisease.org/EN/10.14336/AD.2019.0421

SUPPLEMENTARY DATA

Supplementary Table 1. Search strategy.

Data source	Search terms
**PubMed**	#1 stem cells [Title/Abstract]<br>#2 progenitor cells [Title/Abstract])<br>#3 hematopoietic stem cells [Title/Abstract]<br>#4 mesenchymal stem cells [Title/Abstract]<br>#5 bone marrow mononuclear cells [Title/Abstract]<br>#6 cell therapy [Title/Abstract]<br>#7 #1 OR #2 OR #3 OR #4 OR #5 OR #6<br>#8 diabetes [Title/Abstract]<br>#9 hyperglycemia [Title/Abstract]<br>#10 #8 OR #9<br>#11 #7 AND #10 filters: clinical trial
**Embase**	#1 ‘stem cells’: ti, ab<br>#2 ‘progenitor cells’: ti, ab<br>#3 ‘hematopoietic stem cell’: ti, ab<br>#4 ‘mesenchymal stem cell’: ti, ab<br>#5 ‘bone marrow derived stem cell’: ti, ab<br>#6 ‘cell therapy’: ti, ab<br>#7 #1 OR #2 OR #3 OR #4 OR #5 OR #6<br>#8 ‘diabetes’: ti, ab<br>#9 ‘hyperglycemia’: ti, ab<br>#10 #8 OR #9<br>#11 #7 AND #10<br>#12 #11 AND ('clinical trial'/de OR 'clinical trial (topic)'/de) AND 'article'/it
**The Cochrane Library**	#1 (diabetes):ti, ab, kw OR (hyperglycemia):ti, ab, kw (Word variations have been searched)<br>#2 (stem cells):ab OR (hematopoietic stem cells):ti, ab, kw OR (mesenchymal stem cells):ti, ab, kw OR (bone marrow mononuclear cells):ti, ab, kw AND (cell therapy):ti, ab, kw (Word variations have been searched)<br>#3 #2 AND #1 in Trials
**ClinicalTrials.gov**	“diabetes” AND “stem cells” |studies with Results

© 2019. Zhang Y et al. Published online at http://www.aginganddisease.org/EN/10.14336/AD.2019.0421

SUPPLEMENTARY DATA

Supplementary Table 2. Results of the Downs and Black methodological quality assessment.

Study	Items																											
	1	2	3	4	5	6	7	8	9	10	11	12	13	14	15	16	17	18	19	20	21	22	23	24	25	26	27	Total Score
Ye 2017	1	1	1	1	1	1	1	0	1	0	0	1	1	0	0	1	1	1	0[a]	1	0[a]	0[a]	0	0	0[a]	1	5	20
D'Addio 2014	1	1	1	1	1	1	0	1	1	0	0	1	1	0	0	1	1	1	0[a]	0	0	1	0	0	0[a]	1	5	20
Zhang 2012	1	1	1	1	0	1	1	0	1	1	0	1	1	0	0	1	1	1	0[a]	1	1	1	0	0	0[a]	1	5	22
Li 2012	1	1	1	1	1	1	1	1	1	1	0	1	1	0	0	1	1	1	0[a]	1	1	0	0	0	0[a]	1	5	23
Gu 2012	1	1	1	1	1	1	1	1	1	0	0	1	1	0	0	1	1	1	0[a]	1	0	0	0	0	0[a]	1	5	21
Snarski 2010	1	1	1	1	0	1	1	1	1	0	0	1	1	0	0	1	1	1	0[a]	1	0	1	0	0	0[a]	1	4	20
Couri 2009	1	1	1	1	1	1	1	1	0	1	0	1	1	0	0	1	0	1	0[a]	1	1	0	0	0	0[a]	0	5	20
Voltarelli 2007	1	1	1	1	1	1	1	1	0	1	0	1	1	0	0	1	0	1	0[a]	1	0	0	0	0	0[a]	1	5	20
Carlsson 2014	1	1	1	1	1	1	1	1	0	0	0	1	1	0	0	1	1	1	0[a]	1	1	0[a]	1	0	0[a]	1	5	22
Hu 2013	1	1	1	1	0	0	0	1	1	0	0	1	1	1	1	1	1	1	0[a]	0	0[a]	1	1	1	0[a]	1	5	22
Bhansali 2017	1	1	1	1	1	1	1	1	1	1	0	1	1	1	0	1	1	1	0[a]	1	1	0[a]	1	0[a]	0[a]	1	5	25
Hu 2016	1	1	1	1	1	1	0	1	1	0	0	1	1	0	0	1	1	1	0[a]	0	1	1	1	0	0[a]	1	5	22
Skyler 2015	1	1	1	1	1	0	0	1	0	0	0	1	1	1	0	1	1	1	0[a]	0	1	0	1	0[a]	0[a]	1	5	20
Guan 2015	1	1	1	1	1	1	1	1	1	0	0	1	1	0	0	1	1	1	0[a]	1	1	1	0	0	0[a]	1	3	21
Liu 2014	1	1	1	1	1	0	1	1	1	0	0	1	1	0	0	1	1	1	0[a]	0	0[a]	1	0	0	0[a]	1	5	20
Jiang 2011	1	1	1	1	1	1	1	1	1	0	0	1	1	0	0	1	1	1	0[a]	1	1	1	0	0	0[a]	1	5	23
Bhansali 2017	1	1	1	1	1	1	1	1	1	1	0	1	1	0	0	1	1	1	0[a]	1	1	0[a]	0	0	0[a]	1	4	22
Wu 2014	1	1	1	1	1	1	1	1	1	0	0	1	1	0	0	1	1	1	0[a]	1	0[a]	0[a]	1	0	0[a]	1	5	22
Bhansali 2014	1	1	1	1	1	1	1	0	1	1	0	1	1	1	0	1	1	1	0[a]	1	1	1	1	0[a]	0[a]	1	5	25
Hu 2012	1	1	1	1	1	0	1	1	1	1	0	1	1	0	0	1	1	1	0[a]	1	1	1	0	0	0[a]	1	5	23
Bhansali 2009	1	1	1	1	1	1	1	1	1	1	0	1	1	0	0	1	1	1	0[a]	1	0[a]	1	0	0	0[a]	1	5	23

Abbreviations: Items 1–10: Reporting; Items 11–13: External validity; Items 14–20: Internal validity – bias; Items 21–26: Internal validity - confounding (selection bias); Items 27: Power.

0[a]: Item was unable to be determined, scored 0

© 2019. Zhang Y et al. Published online at http://www.aginganddisease.org/EN/10.14336/AD.2019.0421

SUPPLEMENTARY DATA

Supplementary Table 3A. Results of sensitivity analysis of C-peptide levels in patients with T1DM after BM-HSC therapy.

Study	MD 95% CI	p-value	tau^2	I^2
All studies included (random effects model)	0.49 (0.24, 0.74)	<0.001	0.06	93%
**Omitting D'Addio 2014**	**0.38 (0.21, 0.54)**	**0.16**	**0.01**	**42%**
Omitting Snarski 2010	0.47 (0.20, 0.74)	<0.001	0.07	95%
Omitting Voltarelli 2007	0.46 (0.17, 0.75)	<0.001	0.07	95%
Omitting Ye 2017	0.53 (0.25, 0.81)	<0.001	0.06	94%
Omitting Zhang 2012	0.56 (0.36, 0.76)	0.04	0.02	64%

Supplementary Table 3B. Results of sensitivity analysis of HbA1c levels in patients with T1DM after BM-HSC therapy.

Study	MD 95% CI	p-value	tau^2	I^2
All studies included (random effects model)	-4.11 (-5.11, -3.11)	<0.001	0.79	88%
**Omitting Couri 2009**	**4.54 (-5.38, -3.70)**	**0.10**	**0.32**	**56%**
Omitting D'Addio 2014	-4.32 (-6.22, -2.41)	<0.001	2.38	88%
Omitting Snarski 2010	-3.79 (-4.84, -2.74)	<0.001	0.73	90%
Omitting Ye 2017	-3.98 (-5.25, -2.70)	<0.001	1.01	91%

Supplementary Table 4A. Results of sensitivity analysis of C-peptide levels in patients with T2DM after MSC therapy.

Study	MD 95% CI	p-value	tau^2	I^2
All studies included (random effects model)	0.24 (-0.27, 0.76)	0.08	0.11	60%
**Omitting Bhansali 2017**	**0.59 (0.06, 1.13)**	**0.58**	**0.00**	**0%**
Omitting Jiang 2011	0.22 (-0.32, 0.76)	0.04	0.12	76%
**Omitting Liu 2014**	**0.00 (-0.06, 0.07)**	**0.37**	**0.00**	**0%**

© 2019. Zhang Y et al. Published online at http://www.aginganddisease.org/EN/10.14336/AD.2019.0421

SUPPLEMENTARY DATA

Supplementary Table 4B. Results of sensitivity analysis of C-peptide levels in patient T2DM after BM-MNC therapy.

Study	MD 95% CI	p-value	tau^2	I^2
All studies included (random effects model)	0.36 (0.08, 0.64)	<0.001	0.07	81%
Omitting Bhansali 2009	0.34 (-0.06, 0.73)	0.001	0.12	81%
Omitting Bhansali 2014	0.29 (0.02, 0.56)	<0.001	0.06	82%
**Omitting Bhansali 2017**	**0.47 (0.28, 0.66)**	**0.12**	**0.02**	**49%**
Omitting Bhansali 2017	0.41 (0.07, 0.74)	<0.001	0.08	84%
Omitting Wu 2014	0.34 (-0.05, 0.73)	<0.001	0.12	85%

Supplementary Table 4C. Results of sensitivity analysis of HbA1c levels in patients with T2DM after MSC therapy.

Study	MD 95% CI	p-value	tau^2	I^2
All studies included (random effects model)	-1.54 (-2.48, -0.61)	<0.001	0.72	85%
**Omitting Bhansali 2017**	**-1.89 (-2.70, -1.08)**	**0.06**	**0.31**	**64%**
Omitting Hu 2016	-1.37 (-2.49, -0.25)	0.005	0.75	81%
Omitting Jiang 2011	-1.23 (-2.17, -0.29)	<0.001	0.60	87%
Omitting Liu 2014	-1.72 (-3.03, -0.40)	<0.001	1.13	90%

Supplementary Table 4D. Results of sensitivity analysis of HbA1c levels in patients with T2DM after BM-MNC therapy.

Study	MD 95% CI	p-value	tau^2	I^2
All studies included (random effects model)	-0.51 (-1.13, 0.11)	<0.001	0.53	90%
Omitting Bhansali 2009	-0.39 (-1.12, 0.33)	<0.001	0.62	92%
Omitting Bhansali 2014	-0.65 (-1.28, -0.02)	<0.001	0.44	88%
Omitting Bhansali 2017	-0.67 (-1.28, -0.06)	<0.001	0.41	88%
Omitting Bhansali 2017	-0.60 (-1.28, 0.08)	<0.001	0.54	91%
Omitting Hu 2012	-0.34 (-0.97, 0.29)	<0.001	0.43	84%
Omitting Wu 2014	-0.38 (-1.14, 0.37)	<0.001	0.66	92%

© 2019. Zhang Y et al. Published online at http://www.aginganddisease.org/EN/10.14336/AD.2019.0421

SUPPLEMENTARY DATA

Supplementary Table 4E. Results of sensitivity analysis of FPG levels in patients with T2DM after MSC therapy.

Study	MD 95% CI	p-value	tau^2	I^2
All studies included (random effects model)	-0.49 (-2.60, 1.63)	<0.001	3.29	96%
Omitting Bhansali 2017	-1.29 (-2.92, 0.33)	0.03	1.10	79%
**Omitting Hu 2016**	**0.44 (-0.74, 1.62)**	**0.08**	**0.52**	**67%**
Omitting Liu 2014	-0.55 (-3.41, 2.31)	<0.001	4.18	98%

Supplementary Table 4F. Results of sensitivity analysis of FPG levels in patients with T2DM after BM-MNC therapy

Study	MD 95% CI	p-value	tau^2	I^2
All studies included (random effects model)	-0.53 (-1.53, 0.46)	<0.001	1.42	94%
Omitting Bhansali 2009	-0.38 (-1.49, 0.73)	<0.001	1.51	95%
Omitting Bhansali 2014	-0.75 (-1.87, 0.36)	<0.001	1.47	94%
Omitting Bhansali 2017	-0.80 (-1.87, 0.26)	<0.001	1.34	93%
Omitting Bhansali 2017	-0.69 (-1.89, 0.51)	<0.001	1.72	94%
Omitting Hu 2012	-0.25 (-1.13, 0.62)	<0.001	0.84	87%
Omitting Wu 2014	-0.31 (-1.41, 0.80)	<0.001	1.48	94%

© 2019. Zhang Y et al. Published online at http://www.aginganddisease.org/EN/10.14336/AD.2019.0421
